# Bedside clinical assessment of patients with common upper limb tremor and algorithmic approach

**DOI:** 10.2478/abm-2024-0008

**Published:** 2024-04-30

**Authors:** Pattamon Panyakaew, Warongporn Phuenpathom, Roongroj Bhidayasiri, Mark Hallett

**Affiliations:** Chulalongkorn Centre of Excellence for Parkinson’s Disease and Related Disorders, Department of Medicine, Faculty of Medicine, Chulalongkorn University and King Chulalongkorn Memorial Hospital, Thai Red Cross Society, Bangkok 10330, Thailand; Division of Neurology, Department of Medicine, Faculty of Medicine, Chulalongkorn University, Bangkok 10330, Thailand; The Academy of Science, The Royal Society of Thailand, Bangkok 10330, Thailand; Human Motor Control Section, National Institute of Neurological Disorders and Stroke, National Institutes of Health, Bethesda, MD, 20892-1428, USA

**Keywords:** clinical assessment, investigations, neurophysiology, tremor, upper limb tremor

## Abstract

The diagnostic approach for patients with tremor is challenging due to the complex and overlapping phenotypes among tremor syndromes. The first step in the evaluation of tremor is to identify the tremulous movement and exclude the tremor mimics. The second step is to classify the tremor syndrome based on the characteristics of tremor from historical clues and focused examination (Axis 1). Comprehensive tremor examinations involve the assessment of tremor in different conditions (rest, action or mixed, position or task-specific), distribution of tremor (upper limb, lower limb, head, jaw), positive signs for functional tremor (FT) if suspected (distractibility, entrainment, co-contraction), and associated neurological signs including parkinsonism, dystonic posture, cerebellar/brainstem signs, neuropathy, and cognitive impairment. A pivotal feature in this step is to determine any distinct feature of a specific isolated or combined tremor syndrome. In this review, we propose an algorithm to assess upper limb tremors. Ancillary testing should be performed if clinical evaluation is unclear. The choice of investigation depends on the types of tremors considered to narrow down the spectrum of etiology (Axis 2). Laboratory blood tests are considered for acute onset and acute worsening of tremors, while structural neuroimaging is indicated in unilateral tremors with acute onset, nonclassical presentations, and a combination of neurological symptoms. Neurophysiological study is an important tool that aids in distinguishing between tremor and myoclonus, etiology of tremor and document specific signs of FT. Treatment is mainly symptomatic based depending on the etiology of the tremor and the patient’s disabilities.

Tremor, defined as involuntary rhythmic oscillatory movements of a body part produced by alternating or synchronous contractions of reciprocal agonist and antagonist muscles, is the most common movement disorder that neurologists encounter in clinical practice [1,2,3]. According to the 2018 Consensus Statement from the Task Force on Tremor of the International Parkinson and Movement Disorders Society, tremor is currently classified along 2 axes: clinical features that determine tremor syndromes in Axis 1, and underlying etiology in Axis 2 [1]. Careful history-taking and comprehensive physical examination of tremor characteristics and additional neurological signs are essential for the syndromic identification for Axis 1 [4, 5]. The clinical phenotypes of various tremor syndromes are complex and overlapped, often making the diagnosis challenging [6,7,8]. The lack of validated clinical diagnostic criteria and biomarkers for diagnosis of each tremor syndrome adds to the diagnostic difficulty.

This review article will provide a step-by-step clinical approach to common tremor situations that are usually misdiagnosed in real-life practice. The first step is to confirm that the movements are actually tremors and to exclude tremor mimics [9, 10]. The second step is to assess the tremor during rest, postural holding in different positions, kinetic movements, and task-specific situations. Determination of tremor amplitude, frequency, distribution, and evaluation of the associated systemic and neurological signs can lead to categorizing the tremor into an isolated or combined tremor syndrome [1, 2]. Using a pen and paper for spiral, line, and sentence writing can be useful [11]. Ancillary investigations including laboratory testing, neuroimaging, and neurophysiological study would be necessary to diagnose unclear tremor syndromes and search for the specific etiology [1, 10].

## Step 1: Identify the real tremulous movements and differentiate from tremor mimics

The key feature of tremor is involuntary rhythmic oscillatory movement. Rhythmicity means regular and recurrent, while oscillation refers to a back-and-forth movement, resulting in a sinusoidal movement at a joint [2]. One mimic is rhythmic myoclonus, including cortical tremor, frequent asterixis (negative myoclonus), and polyminimyoclonus [10]. Myoclonus is usually manifested as a sudden, irregular, and shock-like jerky movement. However, high-frequency (8–18 Hz) repetitive myoclonus, called cortical tremor, can be a difficult diagnosis [10, 12,13,14]. In addition, some tremors are not totally rhythmic and may present as an irregular movement such as dystonic tremor (DT) (tremor that appears in the dystonic part), Holmes tremor (HT), and tremor with different amplitudes in various situations, which could clinically overlap with myoclonus [7, 15,16,17]. When the clinical evaluation is in doubt, a neurophysiological study with polymyography (poly-EMG) is indicated to determine “rhythmicity of movement” that might favor tremor over myoclonus [9]. Both tremor and cortical myoclonus could be observed during rest, postural, and kinetic, but tremor is not sensitive to touch unlike myoclonus. Tremor can occur at the distal or proximal limbs unilaterally or bilaterally, while myoclonus mostly presents in the distal limb bilaterally or multifocally [18]. Epilepsia partialis continua (EPC) that looks regular and rhythmic at a low frequency (2–3 Hz) could also be misinterpreted as unilateral jerky tremor. The tonic and clonic components, however, are the main characteristics of EPC. EPC is also always observed at rest and its distribution is limited to the distal part of one limb as opposed to tremor (**Table 1**). Electroencephalography (EEG) can be a helpful tool to document the focal epileptiform discharges in EPC [10, 19]. Clonus is a 5–7 Hz rhythmic involuntary movement that typically occurs in response to a suddenly applied passive stretch on the limb. It might mimic action tremor, but clonus only presents during a passive movement or forceful contraction and is associated with hyperreflexia and spasticity [20, 21]. Indeed, the spectrum of hyperkinetic movements are clinically overlapping, starting from the most regular and rhythmic movements in tremor to irregular tremor, high-frequency myoclonus, and irregular jerky movements in typical myoclonus. Myorhythmia is a slow rhythmic repetitive contraction of cranial and limb muscles at low frequency resembling tremor; however, it does not look like a true oscillatory movement as defined in tremor [22]. Neurophysiological tests using an accelerometer and electromyography (EMG) would be able to distinguish myorhythmia from tremor. Myokymia are brief bursts of single motor unit potentials that fire repetitively at regular or irregular intervals. Clinically, it refers to an undulating rippling movement of muscles underneath the skin that would not cause a back–and-forth movement across a joint and could be easily differentiated from tremor [23].

**Table 1.** Differentiation of tremor from tremor mimics

	**Tremor**	**Cortical myoclonus**	**EPC**
Movement	Oscillatory	Shock-like or jerky	Tonic/clonic
		Single or repetitive	Repetitive
Rhythmicity	Regular but sometimes looks irregular in dystonic tremor syndromes or with some variability in amplitude of tremor	Irregular but can be rhythmic with repetitive myoclonus (8–18 Hz)	Regular and rhythmic (2–3 Hz) but can be irregular
Condition	Rest, postural, kinetic	Rest, postural, kinetic	Rest
Stimulus sensitivity	No reflex sensitivity	Can be triggered with sensory stimulation	No reflex sensitivity
Distribution	Bilateral or unilateral	Bilateral, symmetrical	Unilateral
	Distal or proximal	Distal > proximal	Distal > proximal
	Upper limb > lower limb	Upper limb ~ lower limb	Upper limb > lower limb

## Step 2: Classify tremor clinically, based on historical clues and focused examination

The approach to patients with tremor is based on clinical features. History-taking includes age of onset, clinical progression/evolution, activation of tremor during rest, postural, kinetic or certain tasks or positions, body distribution, site of tremor onset, aggravating and relieving factors, family history of tremor and other movement disorders, and associated systemic and neurological illness [1, 20]. The step-by-step tremor examination with provocation tests and specific observations are essential for a syndromic approach (**Table 2**).

**Table 2.** Step-by-step description of tremor examination

**Activation of tremor**	**Position/task**	**Provocation test/Specific observation**	**Associated examination**
**Rest tremor**	**Sitting position** (hands: hands completely relaxed on the lap; legs: foot placed on the floor, supported against gravity) - Semi-prone - Complete prone - Hanging down from the armrests with forearm supported - Hanging down on sides of a chair	**Cognitive test** - Count backwards - Calculation (100-7) **Motor test** - Sequential fingers (1–4) tapping or alternating toes tapping on the contralateral side	Parkinsonism Dystonic posture
	**Supine position** (hands, legs, head) - Hands supported by pillows - Legs supported on the bed - Head supported by pillows **Walking** (hands)	*Observe characteristics of tremor* *Unilateral vs bilateral* *Suppression of rest tremor at the onset of voluntary movements*	

**Postural tremor**	**Sitting position** (hands, legs, head held against gravity) - Outstretched arms with hands pronated and fingers spread - Outstretched arms with hands supinated and fingers spread - Outstretched arms with palms or dorsum of hands facing each other - Wing position (elbows flexed, hands underneath the chin, not touching each other) - Outstretched leg with knee extended - Head unsupported while turning head to the extreme side **Standing position** (legs)	Putting a slip of paper on top of hands (visualize fine tremor)	Parkinsonism Dystonic posture Cerebellar sign Neuropathy
		Putting the weight on the dorsum of hands	
		*Observe *re-emergence* of tremor during postural holding* *Observe position-specific tremor* *Observe null point, sensory trick*	Systemic signs (hyperthyroidism) Kayser-Fleischer (KF) ring
		*Listening to high frequency tremor with stethoscope*	

**Kinetic tremor**	**Performing task** - Finger-to-nose test (hands) - Heel-to-knee test (legs) - Simple maneuvers (pouring water from a glass, drinking water, using utensils) - Writing (spiral, lines, sentences) - Specific tasks (from history) **Action of specific parts of body** - Jaw: open a month - Tongue: protrude a tongue - Voice: Say “Ahh and Eee)	*Observe simple kinetic vs intentional tremor *Observe task-specific*	Parkinsonism Dystonic posture Cerebellar sign Neuropathy
		*Observe axis, size* *Observe occurrence of tremor at rest vs action*	Systemic signs (hyperthyroidism) KF ring

**Specific signs for functional tremor**	Keep the position that elicits tremor		
	- Cognitive test and motor test with the contralateral/less affected side - Tapping with different frequencies with the contralateral/less affected side - Ballistic movements of the contralateral/less affected side - Passively move tremulous limb before the onset of tremor	*Observe distractibility and tremor variability* *Observe *entrainment* (change/adopt the tapping frequency) *Observe a pause of tremor*	
		*Observe co-activation sign*	

### Step 2.1: Define the tremor characteristics

The examination starts with techniques to elicit tremor according to the activation of tremor (see **Supplementary Figure 1** demonstrating the recommended postures for rest, postural and kinetic tremor examinations). At the resting position, the patients should be asked to sit on a chair with armrests and completely relax their hands and arms on their laps in three different positions: semiprone, complete prone, and hanging their hands down from the armrests with forearm supported or hanging down on the sides of a chair [24]. The complete prone position has the highest specificity (98%) to distinguish between Parkinson’s disease (PD) tremor and essential tremor (ET) with rest tremor. The hanging down position has the highest sensitivity (96%) to evaluate tremor intensity in a patient with a clear diagnosis of PD, because the largest amplitude of rest tremor can be elicited in this position [25]. Sitting position with legs and feet placed on the floor, sitting on the bed with legs hanging, and the supine position could also demonstrate rest tremor of the lower limb. Rest tremor can be provoked with a cognitive loading task by asking the patients to count backward or serial 100–7 or with motor co-activation tasks with sequential fingers or toes tapping on the contralateral side. Suppression of rest tremor at the onset of voluntary movement and the re-emergence of tremor should be looked for during changing positions from rest to postural holding since these signs are suggestive of PD tremor [26, 27]. At postural holding, hands, legs, and head are held against gravity in different positions: outstretched arms with hands pronated or supinated and finger spread, outstretched arms with palms facing each other, and wing position (elbows flexed, hands underneath the chin, not touching each other). Finger spreading helps to exclude polyminimyoclonus and determines touch-sensitivity (myoclonus can be aggravated by touch), while the wing position is for evaluation of proximal tremor [2, 4]. Putting a slip of paper on top of the hands can be helpful to identify fine amplitude tremor [28,29,30]. Loading heavy objects on the patient’s hands during postural holding is another bedside technique to demonstrate changes in tremor frequency and amplitude in cases with enhanced physiologic tremor (EPT) and functional tremor (FT) [31, 32]. In postural head tremor, patients should be asked to move their head to the right and left to see if there is a null point where the amplitude of head tremor is minimized [17]. For kinetic tremor, the finger-to-nose and the heel-to-knee tests are commonly used in clinical practice to define simple kinetic tremor and intentional components where tremor occurs or increases when approaching a target [2, 30]. Tremor that might occur during specific tasks such as when using utensils, holding a cup, pouring a cup of water, and using a mobile phone should also be demonstrated during the examination. Each position should be tested for at least 10 s (**Supplementary Video 1**) (with consent from the patient for publication). Clinicians should decide the position where tremor has maximal severity and categorize patients into predominant rest or action tremor groups that can help narrow down the differential diagnosis [5, 10].

**Supplementary Figure 1.** Bedside examination of rest, postural, and kinetic tremor. 1A–1E for rest tremor examination of hands, 1A; semi-prone position, 1B; complete prone position, 1C; hanging on the armrest, 1D; hanging on side, 1E; supine position for head, hands, and legs tremor at rest. 2A–2D for postural tremor examination of hands, 2A; outstretched arms with hands pronated, 2B; outstretched arms with hands supinated, 2C; outstretched arms with palms facing each other, 2D; wing position, 2E for postural legs tremor; outstretched leg with knee extended, 2F for postural head tremor; turning to the extreme sides. 3A–3E for kinetic tremor examination of hands, 3A; finger-to-nose test, 3B; pouring water with each hand, 3C; using utensils with both hands, 3D; spiral writing without arm supported, 3E; line writing, 3F; for action voice tremor; say “Ahh” and “eee” (with consent from the patient for publication).

**Supplementary Video 1.** A step-by-step tremor examination at rest, multiple postures, kinetic movements, and multiple tasks including pouring water, using utensils, spiral drawings, and writing.

**Figure 1.**
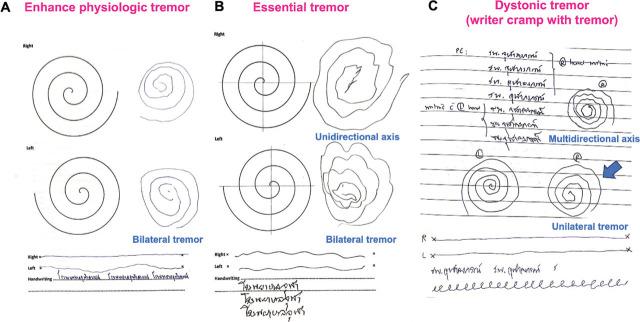
The example of a spiral/line drawing and sentence writing in patients with EPT **(A)**, ET **(B)**, and writer’s cramp with DT **(C)**. EPT and ET show bilateral tremors with spiral drawing with unilateral axis, while DT exhibits tremor on the affected side with multidirectional axis. DT, dystonic tremor; EPT, enhanced physiologic tremor; ET, essential tremor.

Using a pen and paper for writing tasks is a useful bedside examination that may aid in tremor classification. The writing tasks may include asking the patient to draw an Archimedes spiral of at least 10 cm long with hands held off the paper (**Supplementary Video 1**). We can then observe the size of the spiral, tremor frequency/amplitude in the spiral/lines, and the spiral axis [11]. The spiral size would be smaller on the affected side in PD, while there should be no differences between sides in ET and EPT. Tremor could appear unilateral in PD and DT. Handwritten spirals with a unidirectional axis support the diagnosis of ET due to the flexion and extension tremor of the wrist (8 o’clock–2 o’clock on the right; 10 o’clock–4 o’clock on the left). Multidirectional axes and jerkiness of spiral are more suggestive of DT due to the co-contraction of the distal muscles and involvement of the proximal limbs (**Figure 1**). Repetitive drawing could show variability of the spiral in the clockwise and counter-clockwise positions in FT [33, 34]. Repetitive sentence writing (>3 times) can demonstrate micrographia and dystonic posturing during prolonged writing as well as in eliciting mirror dystonia [11].

The second step is to determine the distribution of tremor. Upper limb tremor is often the primary focus during the examination. Tremor in other body parts including face, chin, lip, jaw, voice, head, and legs should also be determined during rest, posture, and kinetic conditions. All body parts should be activated for the evaluation of action tremor such as opening a mouth (jaw), protruding a tongue (tongue), speaking (voice), and standing (legs). Involvement of tremor in certain body parts can point to different tremor syndromes. The details of tremor distribution in each tremor syndrome will be described in the next section.

When FT is suspected from historical clues such as sudden onset with a precise moment, triggered by a minor injury or surgery, fluctuation course, and inconsistency over time, then the specific signs for FT should be determined by an examination [31]. Observation of variability in tremor frequency, amplitude, axis, and distribution are very suggestive of FT [32, 35, 36]. However, frequency variability can also be found in DT [15,16,17]. The combination of tremor variability with other positive clinical signs including distractibility (tremor suppression or attenuation) with cognitive loading or sequential motor tasks, a brief pause of tremor with a contralateral quick movement, entrainment (adopting the tapping frequency) with contralateral tapping with three different frequencies, and tonic coactivation (co-contraction of antagonistic muscle) at the tremor onset are helpful in supporting a diagnosis for FT. However, the sensitivity and specificity of these features may be biased due to the unblinded nature of the assessment in most FT studies [31, 32, 36]. Increased tremor amplitude when weight is applied to the affected limb is another sign of FT. These specific signs for FT should be evaluated to distinguish FT from other tremor syndromes. All details about techniques for tremor examination are listed in **Table 2**.

### Step 2.2: Search for associated neurological signs

The final step for evaluation of tremor is to look for relevant neurological signs associated with tremor. These could be either prominent parkinsonism, cerebellar signs, clear dystonic posture, brainstem signs, and neuropathy or signs of uncertain significance such as mild parkinsonian sign, impaired tandem gait, questionable dystonia, and memory impairment, which could increase diagnostic discrepancies among neurologists [37,38,39,40,41]. Subtle dystonic posture of a body part is often difficult to determine except when it accompanies overflow and mirrors dystonia [42]. The presence of clear associated signs is essential because tremor can be classified into isolated tremor syndrome (tremor occurs in isolation) and combined tremor syndromes where tremor is accompanied by neurological signs. Some tremor syndromes such as HT can present with a combination of cerebellar and brainstem attributes as well as dystonia [43]. Each tremor syndrome in both the isolated and combined groups can be diagnosed based on its clinical features and pattern recognition [6]. Tremor diagnosis can change with time if additional signs develop [7].

## Common tremor situations in clinical practice

### Bilateral action tremor of the upper limb

**Figure 2.**
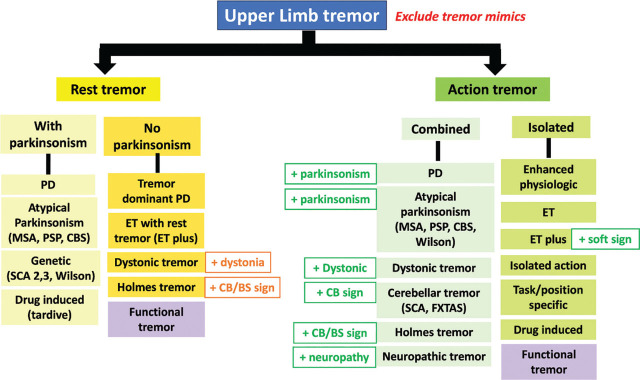
Differential diagnosis of tremor syndromes in the category of action and rest tremor of the upper limb CB; cerebellar, BS; brainstem.

Action tremor of the bilateral upper limbs is one of the most common tremors [10]. For diagnosis, it is important to categorize whether patients have an isolated or combined tremor syndrome (**Figure 2**). If tremor is the only sign, it could be either physiological or pathological tremor depending on the clinical characteristics, distribution, and the frequency of tremor. See below.

#### Isolated action tremor syndromes

1. **Enhanced physiologic tremor (EPT):** Everyone has physiological tremors of bilateral hands due to the mechanical properties of joint and muscles, reflex arc length, and the energy from cardioballistic impulses. If the sensitivity of the stretch reflex is increased from certain medications, thyroid hormone, fatigue, anxiety, or corticospinal lesions, the mechanical tremor will be exacerbated, which is called EPT [44,45,46]. EPT usually has fine amplitude and high frequency at 8–12 Hz and may overlap with ET among young patients [47]. However, tremor in EPT is more prominent in bilateral fingers compared to wrists during postural holding and simple kinetic movements, but there is not an intentional component in contrast to ET (**Table 3**). The head is rarely involved in EPT unlike ET. Putting a sheet of paper on the dorsum of hands will amplify the tremor to make it more obvious [28,29,30]. Tremor frequency can be altered with different tasks and/or postures due to changes in stiffness or contribution of different joints to the tremor [10]. Observations of decreased tremor frequency when heavy objects are loaded on the patient’s hands during postural holding would also point to EPT, since its tremor originates from the peripheral loop of oscillations [44,45,46]. We should almost always look for the precipitating factors including medications such as antiarrhythmics (amiodarone), antidepressants (selective serotonin reuptake inhibitors (SSRIs)/serotonin and norepinephrine reuptake inhibitors (SNRIs), amitriptyline, lithium), anticonvulsants (valproate, lamotrigine), beta-adrenergic agonists (salbutamol), immunosuppressants (tacrolimus, cyclosporine), thyrotoxicosis, and other metabolic disturbances [48].
2. **Essential tremor (ET):** ET is a pathological tremor originating from a central network oscillation in the cerebello–thalamo–cortical pathway, and usually includes recognizable mild cerebellar dysfunction [49]. ET is defined as an upper limb action tremor that has a predominant kinetic nature with an intentional component (42%) usually stronger than with postural holding [50, 51]. ET is usually bilateral but may begin with one side and is mainly exhibited as a flexion and extension of bilateral wrists more than fingers, unlike EPT [1, 7, 30]. ET can subsequently spread to other parts of the body including head, larynx, and lower limbs in longer disease duration, but not isolated head or vocal tremor that is often seen in DT. Tremor frequency does not change with different positions and weight loadings due to it being a central tremor [52]. There may be mild ataxia with intentional tremor that can make it difficult to distinguish ET from cerebellar tremor [51, 53]. Positive family history of action tremor and alcohol responsiveness are clues that can support a diagnosis of ET. Rest tremor may be present at unilateral upper limb in ET but should not be anatomically separated from action tremor with less severity compared to the action component [52, 54]. Rest tremor in ET is often revealed at the flexion and extension of the wrist, which is different from PD tremor (**Table 4**). It is often associated with longer disease duration and requires long-term follow-up with the patient to evaluate the evolution to subsequent diseases [54]. ET is considered a syndrome or a group of diseases since it can have multiple etiologies such as Wilson’s disease or other genetic syndromes (47, XXY; Klinefelter syndrome, 48, XXYY) [55, 56], although it is most commonly idiopathic. It is often necessary to search for the etiology of ET and to follow-up with these patients longitudinally.
3. **Essential tremor-plus (ET-plus):** Patients with ET-plus appear clinically similar to ET but have signs of uncertain significance including mild parkinsonian sign, questionable dystonia, and memory impairment [37,38,39,40,41]. The current controversy is the lack of a definition for these signs particularly subtle dystonia, producing high interrater disagreement in the diagnosis of ET or DT [41]. Spooning (a wrist flexion with hyperextension of fingers) and the index pointing sign (extension of index and partial or full flexion of other digits) are the soft signs of subtle possible dystonia and might therefore support a diagnosis of ET-plus [57, 58]. ET-plus is more common than ET and has an older age of onset with longer disease duration, indicating that ET-plus might be part of a spectrum of ET with advanced stages [59, 60]. In addition, ET-plus could be the state where additional signs emerge during certain stages of ET and may evolve to other diseases [54]. However, it may not be necessary to differentiate ET-plus from ET since both syndromes have similar pathology and similar response to treatment [6, 61, 62].
4. **Intermediate tremor:** Intermediate tremor is the syndrome where bilateral action tremor in the upper limbs only presents during action but is not compatible with the diagnosis of ET or ET-plus due to a duration of <3 years. This category includes tasks or positions specific to when the tremor usually occurs unilaterally without prominent signs of dystonia. Intermediate tremor may evolve to other syndromes [1]. If clear dystonic posture occurs, tasks or positions-specific tremor would become DT. On the other hand, if tremor later develops during postural holding and other types of action, it could evolve to ET with antecedent tasks or positions-specific tremor. In addition, task-specific tremor may be the initial presentation of PD if rest tremor later exhibit at the same side of the task-specific tremor [63].
5. **Drug-induced tremor:** The key in diagnosing drug-induced tremor is the temporal relationship between the offending drugs (as mentioned above in the EPT section) and the occurrence of tremor. This type of tremor has fine amplitude with high frequency and mostly presents at fingers more than at wrists like EPT. However, tremor sometimes occurs at rest if those medications also cause blockages at the dopamine pathway (for example, valproate and amiodarone) [48].

**Table 3.** Clinical features of essential tremor and enhanced physiological tremor

	**Essential tremor**	**Enhanced physiologic tremor**
Activation	Kinetic (intentional component) > postural	Postural or kinetic
	(amplitude of kinetic > postural)	(amplitude of postural > kinetic)
	Rest (in severe cases with action tremor)	Intentional component uncommon
Distribution	Hands (flexion/extension of wrist, bilateral)	Fingers > hands (+/− voice)
	Head, voice, jaw (with hands)	NOT head, jaw
Frequency	4–10 Hz (decrease with age)	8–12 Hz
		Changes with different tasks and different body parts
Amplitude	Fine to large	Fine
Aggravating /relieving factors	Alcohol responsiveness	Increase with stress, exercise, fatigue, caffeine
Pathophysiology	Central oscillator in the cerebello-thalamo-cortical pathway	Sympathetic overactivity and increase sensitivity of peripheral components
Weighting	Similar frequency	Decrease frequency
Treatment	Beta-blocker, primidone, topiramate	Beta-blocker
	Surgery (DBS, MRI FUS thalamotomy)	Decrease aggravating factors
	Assistive devices	

**Table 4.** Clinical characteristics of Parkinson’s disease, dystonic tremor, and essential tremor

	**Parkinson’s disease**	**Dystonic tremor**	**Essential tremor**
Symmetry	Asymmetry	Asymmetry	Symmetry (mostly)
Activation	R > P > K	P ~ K > R	K > P >>> rest
	(Not intentional)	(Not intentional)	(Intentional)
	**Suppression of rest tremor with movements**	Rest (can be early)	Rest (in severe cases with action tremor)
	**Re-emergent tremor**	**position or task specific**	
Distribution	Hands (pill-rolling, rotation of wrist, forearm) > jaw, tongue > leg > head	Head > Hands > jaw Isolated head, voice, jaw	Hands >> head > voice > jaw (with hands in severe cases)
Frequency	4–7 Hz	5–7 Hz	4–10 Hz
Amplitude	Large, waxing & waning	Large, irregular, jerky	Fine to large
Association	Parkinsonism	Dystonic posture	Mild ataxia
	Non-motor symptoms: RBD, anosmia, constipation, depression	Null point (head), overflow/mirror dystonia, sensory trick	Non-motor symptoms: anxiety, depression, cognitive impairment
Family history	Usually absent in late onset	Sometimes present	Present in early onset
Treatment	Dopaminergic agent (response 50%)	Anticholinergics	Beta-blocker, primidone, topiramate
	Anticholinergics	Botulinum toxin injection	Surgery (DBS, MRI FUS thalamotomy)
	Surgery (DBS, FUS)		Assistive devices

DBS; deep brain stimulation, FUS; focus ultrasound, MRI; magnetic resonance imaging, R; rest, P; postural, K; kinetic.

#### Action tremor syndromes combined with other definite neurological signs

**1. With parkinsonism**
  **1.1 Parkinsonian tremor:** Asymmetrical postural tremor with or without rest tremor can be the first manifestation of PD [64]. Postural tremor in PD can be categorized into: (1) re-emergent tremor characterized as a rest tremor that re-emerges after a variable delay while maintaining posture with a frequency similar to the rest tremor [26, 65] and (2) pure postural tremor referring to a postural tremor that presents immediately with postural holding and a tremor frequency higher compared to the rest tremor when it is present [66]. Re-emergent tremor is more common and more specific to PD than pure postural tremor [66]. Pure postural tremor may mimic ET but asymmetrical involvement with associated parkinsonian signs and a presence of non-motor symptoms likely point to PD postural tremor [67]. In addition, kinetic tremor can be observed in 85% of the PD patients with low amplitude and high frequency resembling EPT [9]. Rest tremor occurs in about 65% of the patients with Parkinson’s and increases to a 75% prevalence throughout the course of the disease [9, 68, 69]. It usually presents as a unilateral or asymmetrical rest tremor of the distal upper limb with a frequency of 4–7 Hz. Pill-rolling tremor with flexion beyond the extension of fingers and pronation–supination of the wrist and forearm are the typical characteristics of PD tremor [70]. Suppression of rest tremor at the onset of voluntary movements is very specific to PD tremor [27, 70], particularly when it accompanies re-emergent postural tremor. Rest tremor can be the only presentation that lasts beyond 2 years without other cardinal features of PD, previously known as benign tremulous parkinsonism or monosymptomatic rest tremor, which might evolve to PD [71,72,73]. The presence of resting tremor of jaw, chin, tongue, unilateral leg (thigh abduction/adduction or flexion/extension) or foot (dorsiflexion/plantarflexion) together with unilateral upper limb rest tremor is suggestive of PD (**Table 4**).
  **1.2 Atypical parkinsonism:** Irregular and jerky action tremor is considered one of the red flags for atypical parkinsonian syndrome. Fine amplitude jerky irregular tremor at fingers during postural holding with stimulus-sensitivity known as minipolymyoclonus and intentional tremor may be observed in half of the patients with multiple system atrophy (MSA), while symmetrical action tremor is suggestive of progressive supranuclear palsy (PSP). Rest tremor is less frequent in atypical parkinsonian syndromes. It has been noted in 39% of pathologically proven MSA, more common in MSA-P compared to MSA-C with only 8%–10% observed as pill-rolling tremor [74, 75]. While tremor-at-rest is rarely described in PSP, the parkinsonism subtype (PSP-P) of rest tremor has been reported to occur in about 40% of the patients as an initial presentation indistinguishable from PD [76, 77]. Unilateral/asymmetrical irregular jerky rest and action tremor with dystonic posture of the upper limb was found in 10%–50% of patients with corticobasal syndrome (CBS), which could be DT or myoclonus in nature [78]. Other genetic disorders with parkinsonism that may present with rest tremor include spinocerebellar ataxia (SCA) type 2/3 and Wilson’s disease. However, patients with those genetic disorders generally have both parkinsonism and other cerebellar dysfunctions, pyramidal signs, or dystonia [8].
  **1.3 Drug-induced (tardive) tremor:** Drugs containing a blocking effect on the dopamine pathway such as typical antipsychotic agents or vesicular monoamine transporter type 2 (VMAT2) inhibitors would cause rest tremor resembling PD. However, this type of tremor usually occurs symmetrically and is accompanied by fine-amplitude high-frequency postural tremor and symmetrical parkinsonism [48].
**2. With dystonia:** Tremor has been reported in approximately 50% of the patients with dystonia [79]. Two types of tremors in dystonia have been defined: DT, defined as tremor in the body part affected by dystonia, and tremor associated with dystonia (TAWD), defined as tremor in a body part not affected by dystonia [1]. Both DT and TAWD can present with bilateral upper limb action tremor, mimicking ET/ET-plus.
  **2.1 Dystonic tremor (DT):** DT is a more irregular, jerky, unilateral or asymmetrical tremor that can be position-dependent or task-specific compared to ET [15, 17]. Rest tremor may also be accompanied by action tremor in patients with older age of onset, longer disease duration, and dystonic arm involvement [80,81,82]. It is sometimes challenging to differentiate rest tremor in DT from PD tremor, but thumb extension tremor, rather than pill-rolling tremor, asymmetrical postural tremor without re-emergent pattern, and kinetic tremor are more suggestive of DT [80]. Additional dystonic postures and parkinsonian signs are important clues to distinguish between PD and DT (**Table 4**). Indeed, functional presynaptic dopaminergic imaging may be helpful in the situation where clinical evaluation between two tremor syndromes is still unclear. No dopaminergic deficit is observed in DT, which could be called scans without evidence of dopaminergic deficit (SWEDD) [83]. However, SWEDD is a confusing term that reflects multiple entities, not only DT, and it is unlikely to evolve to PD in the future [84]. Isolated task-specific tremor (e.g., writing, playing instruments) and isolated action of the body segment (isolated head/voice tremor) without postural and kinetic tremor is more suggestive of DT. Examination for significant dystonic posture in the affected body part, overflow, and mirror dystonia produced by contralateral unaffected movements as well as the presence of sensory tricks can be helpful for the diagnosis of DT. The problematic issue with diagnosing DT is due to the unclear definition of clear vs. subtle dystonia, making it challenging to differentiate from ET-plus [38, 40].
  **2.2 Tremor associated with dystonia (TAWD):** Cervical dystonia with hand tremor without dystonic posture of the hand is the most common presentation of TAWD. This subtype of tremor in dystonia is indistinguishable from ET/ET-plus because the tremor has usually been recognized as small, regular, rhythmic, and symmetrical at bilateral hands in TAWD [15].
**3. With cerebellar dysfunction (cerebellar tremor):** Cerebellar tremor is the tremor originating from the cerebellum and cerebellar outflow tracts. Tremor is irregular, low frequency <5 Hz during simple kinetic motion and intentional components accompanied with cerebellar dysfunctions [85]. ET with mild ataxia may look like cerebellar tremor but it is slower and more irregular than ET [4]. Cerebellar tremor can be a manifestation of SCA type 12, type 40 and fragile-X associated tremor and ataxia syndrome (FXTAS) that is usually accompanied by pyramidal signs, parkinsonism, peripheral neuropathy, and cognitive impairment [86].
**4. With neuropathy (neuropathic tremor):** Tremor can develop in neuromuscular disorders including inherited and acquired chronic demyelinating polyneuropathy (CIDP and IgM paraproteinemia), motor neuron diseases, and some myopathic syndromes [87]. Neuropathic tremor is characterized by irregular, jerky, low-frequency (3–6 Hz) action tremor of bilateral upper limbs, predominantly at fingers with abduction and addition rather than flexion and extension tremor of the wrist unlike ET. Tremor in inflammatory neuropathy should also be distinguished from cerebellar tremor due to being predominantly an action tremor with intentional component [88].

#### Tremor with specific clinical features

A combination of rest and action tremor with various severity is one of the most challenging situations that physicians encounter in clinical practice. These types of tremors include specific clinical features with or without the associated neurological symptoms as given below.

**1. Holmes’ tremor (HT):** HT is usually irregular and jerky at a frequency of 1–4 Hz. HT occurs at rest with higher amplitude at postural holding and kinetic movements with intentional components [43]. Both the cerebello–thalamo–cortical pathways and the nigrostriatal pathways are involved in HT. In most patients, HT occurs unilaterally in the upper limbs. Examinations should always look for structural lesions in the thalamus, midbrains, and cerebellum as a part of the Guillain–Mollaret triangle. HT is in the category of combined tremor syndromes that are commonly associated with dystonia, impaired proprioception with pseudoathetoid movements in the thalamic lesion and ataxia, hemiparesis, and cranial neuropathy in midbrain lesion [43, 85, 89]. Cerebellar tremor can look like HT, but no rest tremor is observed in cerebellar tremor. Myorhythmia, defined as slow rhythmic repetitive jerky movements at the 1–4 Hz frequency of cranial and limb muscles with similar amplitude at rest and action, is another movement mimicking HT [22]. Both HT and myorhythmia usually have lesions in the brainstems or thalamus.
**2. Functional tremor (FT):** Patients may present with combined rest tremor and action tremor with similar amplitude but variable tremor frequency, axis, and distribution [32]. Historical clues include sudden onset and a waxing and waning course with spontaneous remission. FT is not a disease of exclusion, but should be ruled in with phenotype-specific signs as mentioned above in the examination section. A combination of distractibility with mental or motor tasks, a brief pause of tremor with a contralateral quick movement, entrainment, suggestibility of tremor with different stimuli by examiners, and tonic coactivation at tremor onset are essential characteristics in supporting a diagnosis for FT [31]. A variable tremor frequency can be observed in other organic tremor syndromes such as DT and EPT with different tasks [16, 90]. However, a mixture of variability of axis and distribution with distractibility is more suggestive of FT.

## Step 3: Are ancillary tests indicated?

Ancillary tests are not indicated if the clinical assessment is clear. The investigations are different depending on the clinical features of the tremor. Blood tests should be indicated if there is an acute onset or acute worsening of tremor or combined tremor syndromes [1, 4, 8, 86]. If there is bilateral upper limb action tremor, metabolic disturbances including thyroid function test, liver or renal function, glucose, and electrolytes may be considered. If tremor is combined with other neurological signs, cognitive or psychiatric problems, or positive family history of movement disorders, serum ceruloplasmin should be tested as a first screen for Wilson’s disease. Other treatable metabolic disorders that may evoke tremor such as Neimann-Pick disease type C, glutaric aciduria type 1, vitamin E deficiency, dopa-responsive dystonia, and coenzyme Q10 deficiency should be considered, if suspected [10].

Neurophysiological testing is a very important tool to confirm the real tremor and search for the etiology of tremor syndromes, but it is not indicated when a diagnosis can be made from clinical assessment [9]. A 1- to 3-axis accelerometer or gyroscope can be used to measure the movement, and multichannel surface EMG (sEMG) can be used to capture the associated motor unit activity [91]. The accelerometers should be placed on the tremulous limb, while the sEMG should be put on the relevant agonist–antagonist muscles [47]. The accelerometer may be an isolated device or can be embedded in a smartphone or smartwatch to objectively evaluate the tremor and quantify tremor severity [92,93,94,95]. The free software (https://www.ifcn.info/software.asp) or ready-to-use software in the tremor devices is used for the analysis of the tremor frequency and amplitude [96]. The first step in neurophysiological testing is to distinguish tremor from tremor mimics, particularly myoclonus [9]. Regular EMG bursts >100 ms in duration with a predominantly alternating EMG pattern between the antagonist pairs are consistent with tremor, while irregular short EMG bursts (<50 ms) standing out from the background with synchronous EMG pattern are suggestive of myoclonus [12, 14]. Irregular, asynchronous, and very short EMG bursts (11–40 ms) would be considered as polyminimyoclonus [97]. The second step is to differentiate between the ET and EPT and document any objective signs of FT [9]. Tremor frequency can overlap in ET and EPT. Demonstration of frequency shifts >1 Hz with weight loading at postural holding with sEMG and accelerometer can discriminate EPT from ET because tremor in EPT comes from the peripheral loop of oscillation [45,46,47]. Suppression of the tremor with cognitive or mental-loading tasks or distractibility and frequency shift to a tapping frequency with contralateral tapping maneuvers or entrainment, a pause in tremor with contralateral ballistic movements, co-activations between the antagonist pairs before the tremor onset, increased tremor amplitude with weight loading, and high coherence between sides if the tremor is bilateral may be easier to document with neurophysiological testing than through a clinical assessment [98]. A combination of these objective signs has been validated as a criteria for diagnosis of FT having a sensitivity of 89.5% and a specificity of 95.9% [99]. Technology-based tremor analysis and machine-learning algorithms can be used to guide the differential diagnosis of tremor syndromes [94]. Technology also can aid to quantitatively measure tremor severity and evaluate responsiveness to treatments.

Structural neuroimaging should be performed in patients with focal or unilateral tremor, nonclassical manifestation, sudden onset or stepwise deterioration, and positive family history of combined tremor syndrome with cognitive or psychiatric problems. Functional imaging such as DaTscan or F-DOPA PET may be considered in distinguishing between ET, DT, or drug-induced parkinsonian tremor and PD patients who would have dopaminergic denervation when the diagnosis is equivocal particularly from neurophysiological studies [10].

Genetic testing can be considered only in combined tremor syndrome with parkinsonism, dystonia, ataxia, neuropathy, cognitive impairment, psychiatric symptoms, and a positive family history of movement disorders [10]. Patients manifesting with bilateral action tremor with ataxia would point to fragile X-associated tremor and ataxia syndrome (FXTAS) or SCA particularly type 12 [100,101,102,103]. In familial cases with dystonia and tremor with or without neuropsychiatric problems, DYT24 (Anoctamin-3 (ANO3) mutation), DYT3 (Lubag’s disease), and Wilson’s disease should be considered [104]. A relevant family history in autosomal dominant with anticipation, autosomal recessive, or X-linked recessive pattern would be essential to suggest different genetic etiologies. Genetic testing can be performed with whole exome sequencing, whole genome sequencing, or targeted resequencing panel.

## Step 4: Do the additional signs develop over time?

The Axis 1 diagnosis of a tremor syndrome can change over time if additional signs develop. For example, the diagnosis would be ET if the patient has bilateral action tremor of the upper limb without other neurological signs for a period of 3 years. Later, if prominent dystonic posture of the head and one arm emerges, the diagnosis could be modified to DT with antecedent ET.

### Treatment

Treatment of tremor depends on the underlying tremor syndrome, tremor-related disability, and the types of tremors as given below.

**1. Enhanced physiologic tremor:** In patients with EPT, treatment or elimination of the reversible causes would be ideal. If patients have tremor due to anxiety, propranolol 10–60 mg or clonazepam or other benzodiazepines prior to the stressful events can be prescribed [86].
**2. Essential tremor:** Medications can reduce the amplitude of limb tremor by 50% in ET, although 50% of the patients fail to respond [56, 105]. Treatment of ET must be individualized and based on patient impairment [106]. The first-line drugs are propranolol and primidone as monotherapy or in combination [105]. Second-line drugs include alprazolam and topiramate (>200 mg/d) [56, 105,106,107,108]. Botulinum toxin injection is appropriate for axial and also for limb tremor [109]. Deep brain stimulation (DBS) at the ventral intermediate nucleus (VIM) may be considered for refractory limb and head tremor with good long-term outcomes [110]. Posterior subthalamic area (PSA) and zona incerta are alternative targets to VIM in ET, which may provide comparable effectiveness for tremor relief with lower currents [111, 112]. Additional stimulations at the Ventral oralis anterior (Voa) or posterior (Vop) nucleus of thalamus with VIM would give more benefit in tremor reduction [111]. MRI-guided focus ultrasound of unilateral thalamotomy is another option without surgery and has shown sustained clinical efficacy in limb tremor [113]. Nonpharmacological treatments including assistive devices and adaptive aids for daily activities have also been found to be helpful [114].
**3. Parkinson’s disease tremor:** Dopaminergic medications may not be effective for PD tremor because the severity of PD rest and action tremor is not correlated to bradykinesia and the index of dopaminergic deficiency [115]. However, levodopa is still useful in some patients. Anticholinergics, amantadine, and clozapine are the other therapeutic options for PD rest tremor, but balancing the benefits with the adverse effects must be considered. The efficacy of DBS at the subthalamic nucleus (STN) and the globus pallidus interna (GPi) have been proven with long-term benefits of up to 10 years for PD tremor [116]. MRI-guided focused ultrasound of unilateral thalamotomy and noninvasive peripheral stimulation are also options for PD tremor [117, 118].
**4. Dystonic tremor:** Anticholinergics, baclofen, benzodiazepine, and beta-blockers can be used. Botulinum toxin injection is the first line for dystonic head tremor [119]. In patients with inadequate response, DBS at GPi would be considered in cases with prominent dystonia, while DBS at VIM would be preferred for marked tremor in dystonia [120].
**5. Holmes’ tremor:** Medications for HT include levetiracetam, trihexylphenidryl, and levodopa, but the response rates to these medications are variable due to a limited number of patients [121]. However, there was about 50% improvement of HT with levodopa in one case series, and the authors suggested that levodopa should be considered in every patient with HT [122]. GPi DBS is preferable compared to VIM DBS if other hyperkinetic movement disorders (chorea/dystonia) are present and the target is not involved by the lesions that are causing HT [121].

## Conclusions

**Figure 3.**
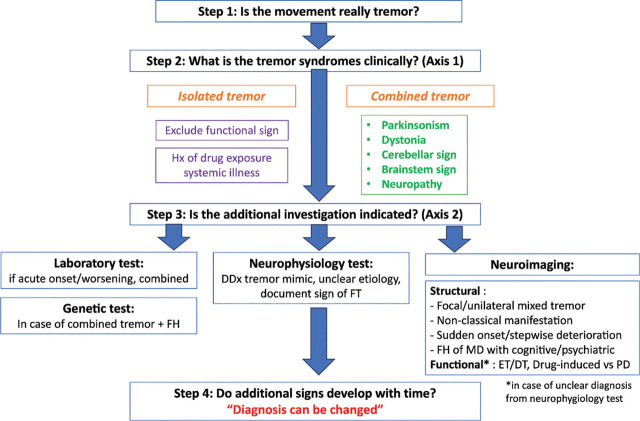
Practical step-by-step algorithms of approaching tremor.

Diagnosis of patients with tremor is challenging due to the complex and overlapping phenotypes. Careful history-taking and focused tremor examination are essential in the exclusion of tremor mimics, evaluation of clinical features, and recognition of specific tremor syndromes. The step-by-step algorithm proposed here for approaching the diagnosis of tremor will make it easier for neurologists who are in clinical practice (**Figure 3**). Treatment should be mainly based on the etiology of tremor and patient’s impairment.

## Supplementary Material

Supplementary Material Details
