# Supplementary figures and images for: Bedside clinical assessment of patients with common upper limb tremor and algorithmic approach
Source: Asian Biomed (Res Rev News). 2024 Apr 30;18(2):37–52. doi: 10.2478/abm-2024-0008 (PMC11063083; doi:10.2478/abm-2024-0008)

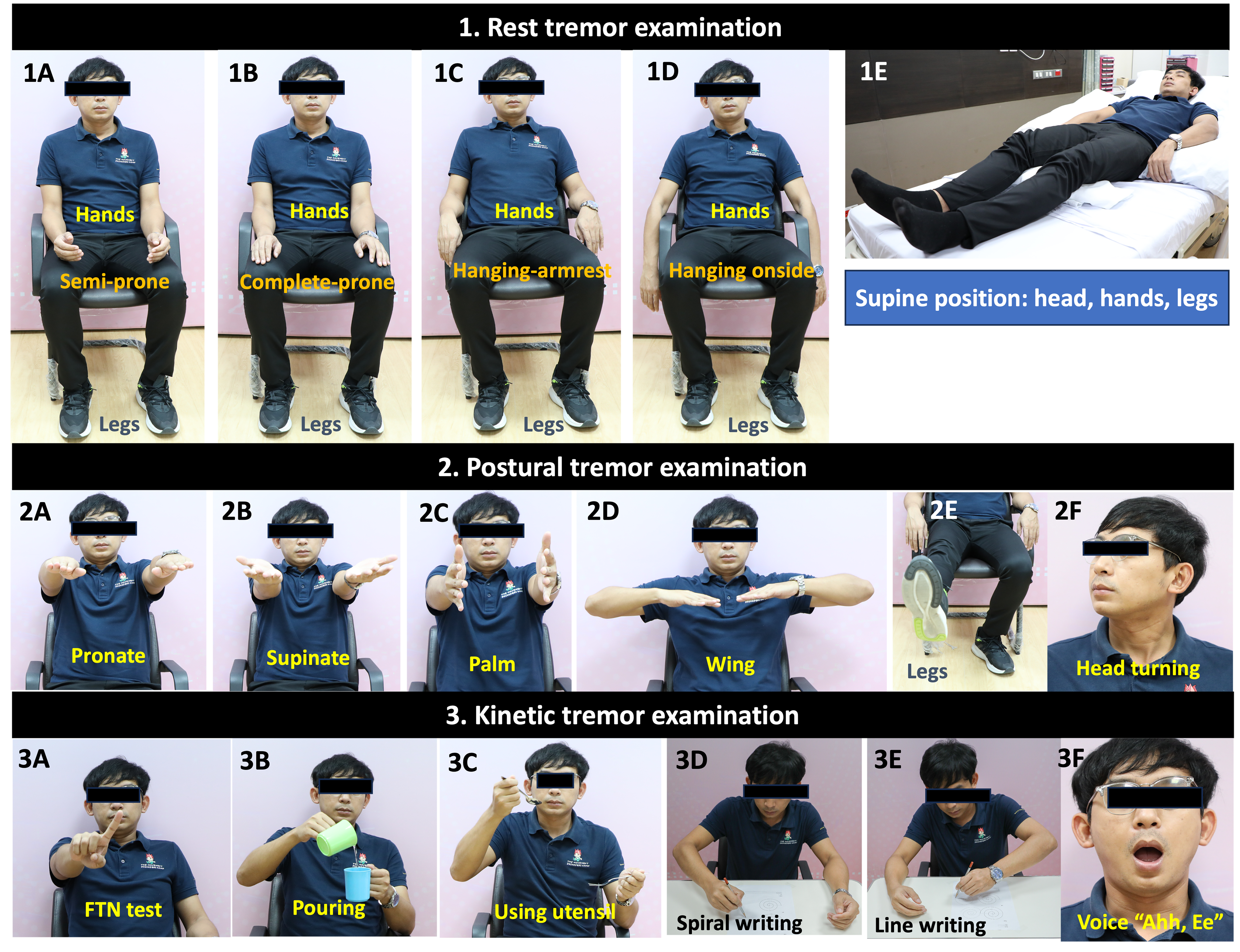

Supplement: Supplementary file 1 — Supplementary Material Details [file abm-2024-0008_sm.zip › Examination.tif]
